# Assessment of First Aid Measures to Control Epistaxis Among Health Care Providers at Tertiary Care Hospital in Saudi Arabia

**DOI:** 10.7759/cureus.28155

**Published:** 2022-08-19

**Authors:** Mazin Merdad, Saad A Sanad, Rakan H Alelyani, Abdulaziz M Alkhammash, Faisal M Swead, Oday M Alghamdi

**Affiliations:** 1 Otolaryngology - Head and Neck Surgery, King Abdulaziz University, Jeddah, SAU; 2 College of Medicine, King Abdulaziz University, Jeddah, SAU

**Keywords:** medical education, health care providers, nose bleed, first aid, epistaxis

## Abstract

Background

Epistaxis refers to bleeding from the inside of the nose or nasal cavity. It is one of the most prevalent otorhinolaryngology emergencies. It is generally treated with simple conservative measures, although it can sometimes be life-threatening. In most cases, using simple first-aid measures that involve tilting the patient's head forward to reduce the risk of blood aspiration and applying digital compression to the nasal alae and anterior septal area for 10-15 minutes will stop the nosebleeds in 90%-95% of cases. This study aims to assess health care providers' knowledge of epistaxis first-aid measures.

Methods

This cross-sectional study was conducted via an electronic questionnaire of multiple-choice questions. The survey was randomly sent through WhatsApp (Google Inc) to all health care providers and medical students who met the inclusion criteria. The study's inclusion criteria included emergency care providers, primary care providers, otolaryngology and head and neck surgery residents, and second-year medical students. In addition, Oto-HNS residents and medical students were included as a control. Respondents were asked to recognize where to apply nasal compression and what is the correct position of the patient's head during an epistaxis episode.

Results

Thirty percent of health care providers answered correctly to the site of nose compression, and 66% for the correct head position. When evaluating the answers to both questions collectively, 31% of EM consultant physicians, 18% of EM residents, 24% of FM consultant physicians, 40% of family medicine residents, 13% of ED nurses, 28% of house officers, 69% of Oto-HNS residents and 17% of the second-year medical students responded to both questions correctly. A large proportion of those who responded incorrectly to either of the questions stated they were “very confident” about their response.

Conclusion

Even though primary and emergency care providers see many epistaxis patients, this study found that the majority of EM consultant physicians, EM residents, ED nurses, FM consultant physicians, FM residents, and house officers surveyed in this study could not identify first-aid measures for epistaxis appropriately. Increased otolaryngology training might help primary and emergency care providers by increasing their understanding of epistaxis first-aid measures often seen in their practices.

## Introduction

Epistaxis is the most common ear, nose, and throat (ENT) condition presented to the emergency department [1]. It is estimated that up to 60% of the general population is affected at some point in their lives, with only about 6% of cases requiring medical intervention [2]. It may lead to severe morbidity, especially in the elderly and those with cardiovascular diseases, or rarely may lead to death [2].

Epistaxis most commonly originates anteriorly from kiesselbach's plexus in 90% of cases; most anterior nosebleeds are self-limiting and do not require medical intervention [3]. In 90%-95% of cases, using simple first-aid measures that involve tilting the patient's head forward to reduce the risk of blood aspiration and applying digital compression to the nasal alae and anterior septal area for 10-15 minutes will stop the nosebleeds [4,5]. Therefore, all health care providers working in the emergency room and those participating in community-based health care should be familiar with epistaxis first-aid methods. Misapplication of first aid management may lead to complications for the patient, such as further blood loss or aspiration if the patient is not in the proper position [6]. Our study aims to assess health care providers' knowledge about first aid measures to control epistaxis at an academic tertiary care center in Saudi Arabia.

## Materials and methods

Study design and data collection

This cross-sectional study was conducted via an electronic questionnaire of multiple-choice questions, evaluating epistaxis first aid management among health care providers at King Abdul-Aziz University Hospital (KAUH). The survey was sent through WhatsApp (Google Inc) to all health care providers and medical students who met the inclusion criteria. The study’s inclusion criteria involved emergency care providers, primary care providers, otolaryngology and head and neck surgery residents, and second-year medical students. We excluded health care providers who are not usually responsible for managing epistaxis or do not work at KAUH. The inclusion criteria were formed to include health care providers that usually encounter epistaxis in an emergency or primary care setting. Data were collected from April 1 through April 30, 2022.

Study participants

We started by sending the questionnaire via WhatsApp to all health care providers who met the inclusion criteria. Then participants were categorized based on their discipline into emergency care providers which included (emergency medicine (EM) consultant physicians, EM residents, emergency department (ED) nurses), primary care providers (family medicine [FM] consultant physicians, FM residents), otolaryngology and head and neck surgery (Oto-HNS) residents, house officers, and second-year medical students. We included house officers and ED nurses as they are usually first-line health care workers encountering epistaxis. The Oto-HNS residents and second-year medical students were incorporated into the study as a control. All participants work at King Abdul-Aziz University Hospital, and the majority of consultants are faculty members at King Abdul-Aziz University (KAU).

Questionnaire variables

The questionnaire has been used in a previous study [7]. To ensure the usability of the questionnaire in our setting, the questionnaire was first tested in our department of otolaryngology and head and neck surgery by distributing it to residents. Residents’ answers were consistently accurate.

**Figure 1:**
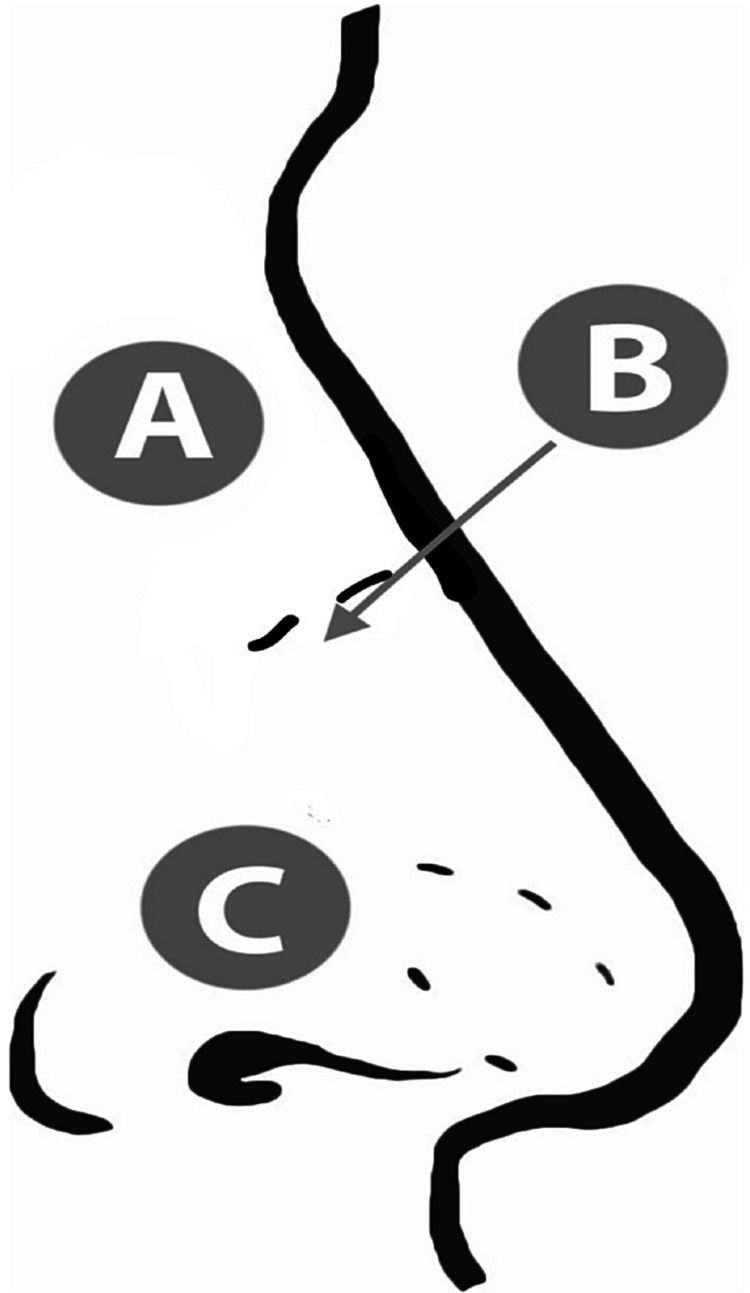
Illustration of the nose for the location of compression

The survey was composed of four questions; two questions about the correct steps of epistaxis management with each question followed by a question about the confidence of their answer. Participants were asked where to apply pressure (the nasal bones, mid-dorsum/rhinion, or nasal ala) and the appropriate position of the head (head neutral, tilted forward, or backward). An image of a nose was provided for the compression site to ensure that participants understood the options (Figure 1). We considered applying pressure to the nasal ala with the head tilted forward as the correct response. In addition, participants were asked how confident they were in each question (unsure, somewhat confident, or very confident).

Statistical analysis

Variables are presented as frequencies and percentages. The analysis was done using IBM SPSS Statistics (Version 25, IBM Corp., Armonk, NY).

Ethical considerations

This study was approved by the Unit of biomedical ethics research committee of KAU, Jeddah, Saudi Arabia reference number (79-22). The contributions were voluntary. Online informed consent was obtained from all participants before starting the survey.

## Results

A total of 131 responses were included for analysis. The respondents were 13 EM consultant physicians, 22 EM residents, 15 ED nurses, 17 FM consultant physicians, 15 FM residents, 18 house officers, 13 Oto-HNS residents, and 18 medical students.

Out of all respondents, only 33.6% correctly identified where to apply compression. 65.6% of respondents correctly positioned the patient’s head. A large proportion of those who responded incorrectly to either of the questions stated they were “very confident” about their response (Table 1). The number of correct responses varied by group, for emergency care providers (Table 2) for primary care providers and house officers (Table 3) for Oto-HNS residents and second medical students (Table 4). When excluding Oto-HNS residents and second-year medical students who were only included in this study for comparative purposes, 30% of respondents correctly identified the site of nasal compression, and 66% had the correct head position. When assessing the responses to both questions together, only 31% of EM consultant physicians, 18% of EM residents, 24% of FM consultant physicians,40% of family medicine residents, 13% of ED nurses, 28% of house officers, 69% of Oto-HNS residents and 17% of second-year medical students responded to both questions correctly.

**Table 1:** Incorrect responders degree of confidence to nasal compression and patient positioning questions during epistaxis

Group (n)	Question with Number of Incorrect Responders (n)	Degree of Confidence		
		Unsure n (%)	Somewhat Confident n (%)	Very Confident n (%)
EM Consultant (13)	Location of Compression (8)	0 (0)	1 (12.5)	7 (87.5)
	Patient position (7)	0 (0)	2 (28.5)	5 (71.5)
EM Resident (22)	Location of Compression (17)	0 (0)	7 (41)	10 (59)
	Patient position (9)	0 (0)	4 (44)	5 (66)
ED Nurse (15)	Location of Compression (12)	1 (8.3)	5 (41.7)	6 (50)
	Patient position (6)	0 (0)	0 (0)	6 (100)
FM Consultant (17)	Location of Compression (13)	0 (0)	5 (38.5)	8 (61.5)
	Patient position (6)	0 (0)	2 (33.3)	4 (66.7)
FM Resident (15)	Location of Compression (7)	1 (14.2)	3 (42.9)	3 (42.9)
	Patient position (5)	1 (20)	2 (40)	2 (40)
House Officer (18)	Location of Compression (13)	2 (15.3)	5 (38.5)	6 (46.2)
	Patient position (1)	0 (0)	0 (0)	1 (100)
Medical Student (18)	Location of Compression (13)	7 (53.8)	4 (30.8)	2 (15.4)
	Patient position (9)	4 (44.4)	5 (55.6)	0 (0)
Oto-HNS Residents (13)	Location of Compression (4)	0 (0)	0 (0)	4 (100)
	Patient position (2)	0 (0)	0 (0)	2 (100)

**Table 2:** Emergency care providers' response regarding the site of nose compression and patient positioning

Group (n)	Site of compression, n (%)	Head position, n (%)
Emergency medicine consultant physicians (13)	Nasal ala 5 (38%)	Forward 6 (46%)
	Mid-dorsum/Rhinion 7 (54%)	Neutral 3 (23%)
	Nasal bone 1 (8%)	Backward 4 (31%)
Emergency medicine residents (22)	Nasal ala 5 (23%)	Forward 13 (59%)
	Mid-dorsum/Rhinion 14 (63%)	Neutral 3 (14%)
	Nasal bone 3 (14%)	Backward 6 (27%)
Emergency department nurses (15)	Nasal ala 3 (20%)	Forward 9 (60%)
	Mid-dorsum/Rhinion 7 (47%)	Neutral 2 (13%)
	Nasal bone 5 (33%)	Backward 4 (27%)

**Table 3:** Primary care providers' response regarding the site of nose compression and patient positioning

Group (n)	Site of compression, n (%)	Head position, n (%)
Family medicine consultant physicians (17)	Nasal ala 4 (23%)	Forward 11 (65%)
	Mid-dorsum/Rhinion 11 (65%)	Neutral 5 (29%)
	Nasal bone 2 (12%)	Backward 1 (6%)
Family medicine residents (15)	Nasal ala 8 (53%)	Forward 10 (67%)
	Mid-dorsum/Rhinion 6 (40%)	Neutral 4 (27%)
	Nasal bone 1 (7%)	Backward 1 (6%)
House officers (18)	Nasal ala 5 (28%)	Forward 17 (94%)
	Mid-dorsum/Rhinion 12 (67%)	Neutral 0 (0%)
	Nasal bone 1 (5%)	Backward 1 (6%)

**Table 4:** Otolaryngology and head and neck surgery residents and second-year medical students responses regarding the site of nose compression and patient positioning

Group (n)	Site of compression, n (%)	Head position, n (%)
Otolaryngology and head and neck surgery residents (13)	Nasal ala 9 (69%)	Forward 11 (85%)
	Mid-dorsum/Rhinion 4 (31%)	Neutral 2 (15%)
	Nasal bone 0 (0%)	Backward 0 (0%)
Second year medical students (18)	Nasal ala 5 (28%)	Forward 9 (50%)
	Mid-dorsum/Rhinion 12 (67%)	Neutral 4 (22%)
	Nasal bone 1 (5%)	Backward 5 (28%)

## Discussion

The responders in this study were EM consultants, EM residents, ED nurses, FM consultants, FM residents, and house officers. Oto-HNS residents and medical students were included as a control. This study demonstrates that epistaxis first-aid measures knowledge among health care providers is poor. Only 30% identified the correct steps of epistaxis first-aid measures. Additionally, a large number of participants stated that they were “very confident” in their incorrect responses. This implies that these physicians are not simply unaware of the epistaxis first aid measures but may have long-held misconceptions about how epistaxis should be treated. A prior assessment of otolaryngology training at US medical schools found substantial variation in medical students' exposure to the field, with just a few institutions offering mandatory rotations [8].

Unfortunately, these findings are not exactly a surprise. Previous studies by Alyahya et al. and Abu-Zaid et al. to assess medical students’ knowledge of epistaxis first aid management found that 41% and 52% identified the correct site for nasal compression respectively, with the majority of participants reporting that knowledge is self-taught [9,10]. Another study conducted in the United Kingdom showed over half of the junior ED residents have not been formally taught to manage epistaxis and 38% had it covered in less than 15 minutes [11].

Patient education is pivotal in today’s medical practice as patients are getting more involved in their medical care [12]. To do so, health care providers should have a sufficient and accurate source of information. A previous study showed the importance of involving Oto-HNS physicians for epistaxis. Patients who were seen by otolaryngologists were less likely to present back with complications and were well-educated about self-first aid measures. The study also found that while all patients had seen a physician prior to seeing Oto-HNS physicians, only about half had received any advice about first-aid management, and those who did were more likely to identify proper measures [13]. Family medicine residents were the best at identifying the proper steps in our study, with only 40% of them able to answer both questions correctly. In comparison to the study done by Sowerby et al., which reveals that only 21% of EM attending physicians identified the correct first aid measures for epistaxis and were better than other health care providers [7]. We believe that family medicine residents got the best score because part of their residency training is to have a mandatory Oto-HNS rotation, unlike emergency care providers. To improve patient education and quality of care, we have to ensure that the education of health care workers is sufficient.

This study has its inherent limitation. It was conducted at only a single institution. We must be careful not to generalize these findings to other health care providers since the data might indicate a deficiency specific to this institution.

## Conclusions

Even though primary and emergency care providers see many epistaxis patients, this study found that the majority of EM consultant physicians, EM residents, ED nurses, FM consultant physicians, FM residents, and house officers surveyed in this study could not identify first-aid measures for epistaxis appropriately. Increased otolaryngology training might help primary and emergency care providers by increasing their understanding of epistaxis first-aid measures.

## Appendices

1.   In case of epistaxis where to apply pressure on the picture below?

A.    The nasal bones

B.    Mid-dorsum/rhinion

C.    Nasal ala

How confident are you?

A.    Very confident

B.    Somewhat confident

C.    Unsure

2.    How the patient’s head should be positioned?

A.    Head neutral

B.    Tilted forward

C.    Tilted backward

How confident are you?

A.    Very confident

B.    Somewhat confident

C.    Unsure
